# Tetramethylpyrazine Prevents Contrast-Induced Nephropathy via Modulating Tubular Cell Mitophagy and Suppressing Mitochondrial Fragmentation, CCL2/CCR2-Mediated Inflammation, and Intestinal Injury

**DOI:** 10.1155/2019/7096912

**Published:** 2019-05-16

**Authors:** Xuezhong Gong, Yiru Duan, Junli Zheng, Zi Ye, Tom K. Hei

**Affiliations:** ^1^Department of Nephrology, Shanghai Municipal Hospital of Traditional Chinese Medicine, Shanghai University of Traditional Chinese Medicine, 274 Zhijiang Middle Road, Shanghai 200071, China; ^2^Center for Radiological Research, Department of Radiation Oncology, College of Physician and Surgeons, Columbia University, 630 West 168th Street, NY 10032, USA

## Abstract

Contrast-induced nephropathy (CIN) is a leading cause of hospital-acquired acute kidney injury (AKI), but detailed pathogenesis and effectual remedy remain elusive. Here, we tested the hypothesis that contrast media (CM) impaired mitochondrial quality control (MQC) in tubules, including mitochondrial fragmentation and mitophagy, induced systemic inflammation, and intestinal injury. Since we previously demonstrated that the natural antioxidant 2,3,5,6-tetramethylpyrazine (TMP) can be a protectant against CIN, we moreover investigated the involved renoprotective mechanisms of TMP. In a well-established CIN rat model, renal functions, urinary AKI biomarkers, and renal reactive oxygen species (ROS) production were measured. Mitochondrial damage and mitophagy were detected by transmission electron microscopy (TEM) and western blot. The abundance of Drp1 and Mfn2 by western blot and immunohistochemistry (IHC) was used to evaluate mitochondrial fragmentation. TUNEL staining, TEM, and the abundance of cleaved-caspase 3 and procaspase 9 were used to assay apoptosis. We demonstrated that increased mitophagy, mitochondrial fragmentation, ROS generation, autophagy, and apoptosis occurred in renal tubular cells. These phenomena were accompanied by renal dysfunction and an increased excretion of urinary AKI biomarkers. Meanwhile, CM exposure resulted in concurrent small intestinal injury and villous capillary endothelial apoptosis. The abundance of the inflammatory cytokines CCL2 and CCR2 markedly increased in the renal tubules of CIN rats, accompanied by increased concentrations of IL-6 and TNF-*α* in the kidneys and the serum. Interestingly, TMP efficiently prevented CM-induced kidney injury *in vivo* by reversing these pathological processes. Mechanistically, TMP inhibited the CM-induced activation of the CCL2/CCR2 pathway, ameliorated renal oxidative stress and aberrant mitochondrial dynamics, and modulated mitophagy in tubular cells. In summary, this study demonstrated novel pathological mechanisms of CIN, that is, impairing MQC, inducing CCL2/CCR2-mediated inflammation and small intestinal injury, and provided novel renoprotective mechanisms of TMP; thus, TMP may be a promising therapeutic agent for CIN.

## 1. Introduction

Contrast-induced nephropathy (CIN) has become the third most common cause of hospital-acquired renal failure, affecting as many as 25% of susceptible patients because of the increasing use of iodine contrast media (CM) after angiocardiography or radiological procedures in clinics [1]. However, the pathogenesis of CIN is still not fully understood, and as a result, there is still a lack of effective strategies for the prevention and mitigation of CIN [2]. Hence, there is an urgent need for novel therapeutic regimens for the clinical management of CIN.

Previous studies by our laboratory and other investigators have demonstrated that oxidative stress due to the overproduction of free radicals induces the apoptosis of renal cells and represents an important pathogenic mechanism in CIN [3–5]. As highly dynamic organelles that undergo frequent movement (fission and fusion), mitochondria are the major source of cellular reactive oxygen species (ROS) as well as a major target of ROS-induced damage [6]. Dysfunctional mitochondria may exhibit fragmentation and membrane depolarization, and these events induce further ROS production [7]. Renal proximal tubular cells are rich in mitochondria, and thus, tubules are vulnerable to mitochondrial damage [8]. The process of removing damaged mitochondria through autophagy, which is termed mitophagy, has recently been proposed to be critical in ischemia and drug-induced tissue damage [6]. Furthermore, mitophagy and mitochondrial fragmentation are critical components of the intracellular mitochondrial quality control (MQC) machinery [9–11]. Thus, we speculated that although the roles of mitophagy induction and mitochondrial fragmentation in CIN remain unknown, mitophagy may be involved in the pathogenesis of CIN. Previous work from our laboratory has shown that autophagy requires the activity of the protein Drp1 [12, 13], which was used as a marker of autophagy in this study.

ROS and inflammation are tightly linked in both acute kidney injury (AKI) and chronic kidney disease [14–16]. Inflammation might play a crucial role in the pathogenesis of CIN, but the underlying molecular mechanisms remain largely unknown [17, 18]. As a member of the C-C chemokine family, CCL2, which is also known as MCP-1, is an important inflammatory mediator, and chemokine receptor 2 (CCR2) could act as its major specific receptor [19, 20]. Recent studies have indicated that CCL2/CCR2-mediated inflammation plays crucial roles in regulating tubular damage in both renal ischemia and reperfusion AKI as well as some experimental models of chronic tissue injury [20, 21]. However, the role of CCL2/CCR2 signaling in the pathogenesis of CIN has not yet been clarified and was therefore investigated in this study.

There is evidence from animal studies that AKI is a multisystem disease affecting the lung, heart, liver, and intestine [22–25]. However, to our knowledge, potential extrarenal complications in CIN have not been previously studied. Thus, another focus of the present study was to elucidate the link between CIN and intestinal injury using an *in vivo* model of CIN.

2,3,5,6-Tetramethylpyrazine (TMP) is an active ingredient of the Chinese herb *Ligusticum wallichii* Franch and has been found to function as a vasodilator, a free radical scavenger, and a suppressor of inflammation [26–28]. It is used in traditional Chinese medicine to protect the kidney from tubular damage caused by gentamicin, diabetes, and ischemia/reperfusion [14, 29, 30]. Our previous studies clearly highlighted that TMP might be a new potential therapeutic agent to prevent CIN by inhibiting p38 MAPK and FoxO1 pathways and could protect renal tubular cells against arsenite-induced nephrotoxicity by preventing mitochondrial dysfunction and autophagy [14, 31]. We thus further speculated that TMP might modulate MQC in tubules.

In the present study, we aimed to test the hypothesis that CM impaired MQC in tubules, including mitochondrial fragmentation and mitophagy; induced CCL2/CCR2-mediated systemic inflammation and intestinal injury; and investigated the involved renoprotective mechanisms of TMP against CIN.

## 2. Materials and Methods

### 2.1. Reagents

All chemicals were purchased from Sigma-Aldrich (St. Louis, MO, USA) unless otherwise stated. The CM iohexol (Omnipaque) was purchased from Amersham Health (Princeton, NJ, USA).

### 2.2. Animals

All studies involving animals were previously reviewed and approved by the Animal Welfare and Ethical Use Committee of the Shanghai Municipal Hospital of Traditional Chinese Medicine. Thirty-two adult 8–10-week-old male Sprague Dawley rats weighing 200-250 g were purchased from the Shanghai Laboratory Animal Research Center (certificate number: 2017-010). The rats were housed in an air-conditioned room at 23°C with a cycle of 12 h/12 h light/dark. Food and water were provided *ad libitum* except for the day of dehydration treatment.

### 2.3. Experimental Protocol and Drugs

A well-established rat model of CIN was used [3, 32–34]. Briefly, rats were randomly assigned into 4 groups of 8 animals each: controls (Con), rats injected with CM (CIN), rats treated with 150 mg/kg/d *N*-acetylcysteine (NAC) and injected with CM (CIN+NAC), and rats treated with 80 mg/kg/d TMP and injected with CM (CIN+TMP). TMP and NAC were injected intraperitoneally (ip) once daily for 4 consecutive days (days 1-4). The Con and CIN groups were administered with the same volume of saline. On day 3, all rats were kept without water for 24 h. On day 4, 20 min after injecting saline, NAC, or TMP, the CIN, CIN+NAC, and CIN+TMP groups were injected with N(G)-nitro-ʟ-arginine methyl ester (10 mg/kg, ip). Then, after a further 15 and 30 min, respectively, they were injected with indomethacin (10 mg/kg, ip) and iohexol (1.5-2 g iodine/kg, ip). The Con group received an equivalent volume of saline by ip injection. On day 5, all rats were provided with regular chow and tap water in metabolic cages for 24 h.

Twenty-four-hour urine samples were collected in metabolic cages on days 1 (baseline) and 5 for the determination of urinary *N*-acetyl-*β*-glucosaminidase (UNAG) and urinary *γ*-glutamyl transpeptidase (UGGT). Baseline blood samples were taken under ether anesthesia from the tail vein for the measurement of serum creatinine (Scr), blood urea nitrogen (BUN), and plasma cystatin-C (CysC). At the end of day 5, blood samples were taken from the abdominal aorta under pentobarbital (50 mg/kg) anesthesia for the measurement of Scr, BUN, and CysC. Subsequently, the rats were euthanized, and the kidneys were collected for biochemical and morphological examinations.

## 3. Histopathological Examinations

### 3.1. Light Microscopy

Tissue samples from the left kidney were fixed in 10% formalin and prepared for examination by light microscopy using either hematoxylin and eosin staining or immunohistochemistry (IHC).

IHC was performed as previously described [3, 14, 31]. Briefly, tissue sections (4 mm) were incubated for 1 h at room temperature with one of the following primary antibodies: anti-Drp1, Abcam (Cambridge, UK), 1 : 500; anti-Mfn2, Abcam, 1 : 500; anti-CCL2, Cell Signaling Technology, 1 : 1000; and anti-CCR2, Cell Signaling Technology, 1 : 1000. Immunodetection was performed using an appropriate biotinylated immunoglobulin and a horseradish peroxidase-labeled avidin kit (Zhongshan Golden Bridge Biotechnology, Beijing, China) with diaminobenzidine as the substrate. Finally, the slides were lightly counterstained with hematoxylin for 30 s. The positive signals were measured using a Motic Med 6.0 CMIAS image analysis system (Motic China Group, Xiamen, China). The staining intensity was measured in a double-blinded manner by two independent investigators and was calculated as the ratio of the stained areas over the total analyzed fields. Ten randomly chosen fields per section were analyzed.

### 3.2. Transmission Electron Microscopy (TEM)

Right renal cortex samples were divided into pieces (2 × 2 mm) on ice, fixed in 2.5% (*v*/*v*) glutaraldehyde-polyoxymethylene solution for 6–8 h at 4°C, and then embedded in Epon® 812 resin. Ultrathin sections (60–70 nm) were stained with uranyl acetate and alkaline lead citrate and visualized under a JEM 100CX transmission electron microscope (JEOL Ltd., Tokyo, Japan) to perform ultrastructural analyses and evaluate autophagy as well as mitophagy. The number of autophagic vacuoles per tubular cell was counted.

### 3.3. Measurement of ROS Generation, IL-6, TNF-*α*, and Terminal Deoxynucleotidyl Transferase dUTP Nick End Labeling (TUNEL) Staining

The oxidation of 2′,7′-dichlorofluorescin diacetate (DCFDA, Jiancheng Research Institute, China) was used to detect intracellular ROS in whole kidney homogenates. Briefly, whole kidney homogenates were incubated with fresh DCFDA (10 *μ*mol/L) for 30 min at 37°C. The absorbance of DCFDA fluorescence at 485 nm was measured against that at 525 nm as the reference wavelength. ROS production was expressed in arbitrary units corrected for protein concentration (AU/mg protein). The concentrations of IL-6 and TNF-*α* in supernatants from renal tissue homogenates and sera were assessed using enzyme-linked immunosorbent assay kits (Abcam, UK) in accordance with the manufacturer's instructions. All assays were performed in duplicate. For TUNEL staining, an *in situ* cell death detection kit (Roche Applied Science, Penzberg, Germany) was used according to the manufacturer's instructions. TUNEL-positive tubular cell numbers were counted randomly in 20 nonoverlapping cortical fields under 400x magnification.

### 3.4. Quantitative Polymerase Chain Reaction (qPCR)

Renal cortexes were dissected and total RNA was extracted using TRIzol® reagent according to the manufacturer's instructions (Invitrogen, Carlsbad, CA). cDNA was synthesized using random primers and a high-capacity cDNA reverse transcription kit (Applied Biosystems, Foster City, CA). The following primers were used: *HO-1* F-TTAAGCTGGTGATGGCCTCC, R-GTGGGGCATAGACTGGGTTC; *NQO1* F-GTTTGCCTGGCTTGCTTTCA, R-ACAGCCGTGGCAGAACTATC; and *beta-actin* F-CTGTGTGGATTGGTGGCTCT, R-GCAGCTCAGTAACAGTCCGC. qPCR was performed on a 7500 real-time PCR system (Applied Biosystems).

### 3.5. Western Blot Analysis

Western blotting was performed as previously described [3, 5]. The primary antibodies used were as follows: anti-LC3B (Cell Signaling Technology, Danvers, MA), 1 : 1000; anti-beclin-1 (Cell Signaling Technology, Danvers, MA), 1 : 1000; anti-CCL2 (Abcam, UK), 1 : 1000; anti-CCR2 (Abcam, UK), 1 : 1000; anti-p62 (Abcam, UK), 1 : 1000; anti-Drp1 (Abcam, UK), 1 : 1000; anti-Mfn2 (Abcam, UK), 1 : 1000; anti-procaspase 9 (Cell Signaling Technology, Danvers, MA), 1 : 1000; anti-cleaved caspase 3 (Cell Signaling Technology, Danvers, MA), 1 : 1000; anti-histone (Cell Signaling Technology, Danvers, MA), 1 : 2000; and anti-GAPDH (Cell Signaling Technology, Danvers, MA), 1 : 5000. All experiments were performed at least three times (i.e., three separate protein preparations) under the same conditions.

### 3.6. Statistical Analysis

Results are expressed as means ± SD. One-way analysis of variance (ANOVA) with Tukey's post hoc multiple-comparison test was used to determine the significance of differences in multiple comparisons. Differences were considered significant if *p* < 0.05 and highly significant if *p* < 0.01.

## 4. Results

### 4.1. TMP Protected the Kidney from Damage in a Rat CIN Model

**Figure 1:**
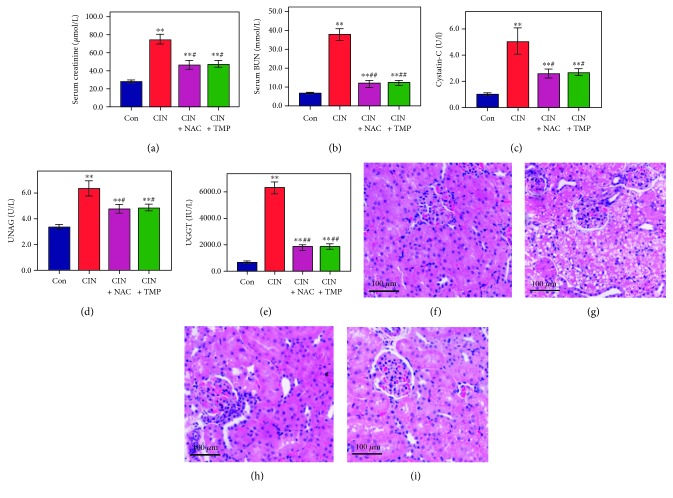
Tetramethylpyrazine (TMP) protected the kidney from damage in a rat contrast-induced nephropathy (CIN) model. The serum levels of creatinine (a), blood urea nitrogen (b), cystatin-C (c), urinary *N*-acetyl-*β*-glucosaminidase (d), and urinary *γ*-glutamyl transpeptidase (e) were examined using automated biochemistry assays. Photomicrographs (original magnification, ×200) illustrate hematoxylin and eosin staining of the kidney tissues from rats in the following groups: control (Con; (f)), CIN without treatment (CIN; (g)), CIN with *N*-acetyl cysteine treatment (CIN+NAC; (h)), and CIN with TMP treatment (CIN+TMP; (i)). Figures are representative of 5–8 rats from each group. Data are represented as means ± standard deviation (SD; *n* = 5). ^∗∗^*p* < 0.01 versus Con and ^##^*p* < 0.01 versus CIN.

As shown in Figure 1, the levels of Scr, BUN, CysC, UNAG, and UGGT in the CIN group were markedly elevated compared with those in the Con group at day 5 (*p* < 0.01). Pretreatment with either TMP or NAC significantly suppressed the levels of Scr, BUN, CysC, UNAG, and UGGT in the CIN rats (*p* < 0.01) with similar activities (Figure 1).

Histologic analysis demonstrated a normal glomerulus structure in the Con animals (Figure 1(f)), while severe renal tubular interstitial injury was observed in the kidneys of the CIN-treated rats, including obvious vacuolar degeneration of tubular epithelial cells, loss of the tubular brush border, increased epithelial cell shedding, and necrosis of tubular epithelial cells (Figure 1(g)). These signs of tissue damage were markedly relieved by treatment with TMP and NAC (Figures 1(h) and 1(i), respectively).

### 4.2. TMP Modulated Renal Tubular Cell Autophagy, Mitophagy, and Apoptosis in the Kidneys of CIN Rats

**Figure 2:**
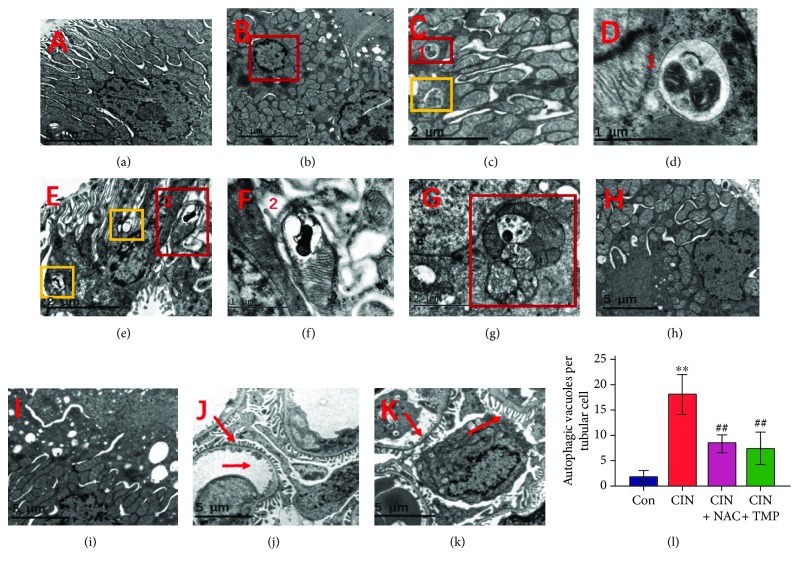
Tubular epithelial cell mitophagy and autophagy were increased in CIN rats and suppressed by TMP pretreatment. Transmission electron microscopy (TEM) images of the kidney in the Con (a), CIN (b–g), CIN+NAC (h), and CIN+TMP (i) rats. The red frame in (b) indicates renal tubular cells undergoing apoptosis in CIN rats as characterized by the condensation of nuclear chromatin. TEM of autophagosomes in CIN rats (orange frames in (c) and (e), indicative of autophagy) and mitochondria encapsulated in autophagosomes in CIN rats (red frames in (c), (e), and (g), indicative of mitophagy) (×9000). (d) Higher magnification TEM of (c) (×27000). (f) Higher magnification TEM of (e) (×27000). Red arrows indicate the normal glomerular basement membrane and podocytes in CON rats (j) and CIN rats (k) (×4200). (l) Quantification of the numbers of autophagic vacuoles in each tubular cell. ^∗∗^*p* < 0.01 versus Con and ^##^*p* < 0.01 versus CIN.

We performed ultrastructural analyses to ascertain the presence of renal tubular programmed cell death (autophagy and apoptosis) as well as mitophagy in CIN rats using TEM. As shown in Figures 2(c) and 2(e), renal tubular autophagy in CIN kidneys after 24 h of CM injection was characterized by higher quantities of autophagosomes (indicated by yellow or red frames in Figures 2(c), 2(e), and 2(l)) and double or multiple membrane-encapsulated components when compared to that in control kidneys. These results indicated that CM induced renal tubular cell autophagy in rats.

**Figure 3:**
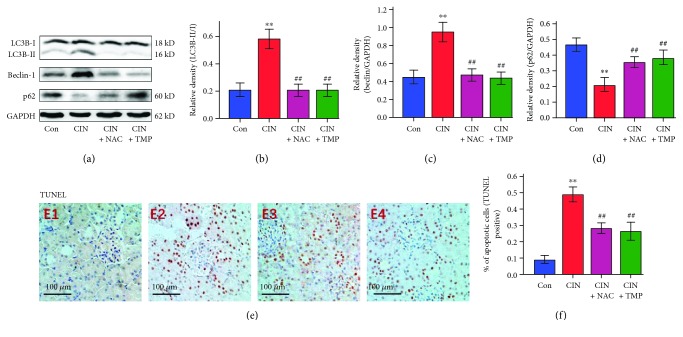
Analysis of LC3B, beclin-1, and p62 protein abundance and terminal deoxynucleotidyl transferase dUTP nick end labeling (TUNEL) staining. The autophagy-associated proteins LC3B and beclin-1 were detected by western blotting (a). The abundance of LC3B-II (b), beclin-1 (c), and p62 (d) was quantified by densitometry and normalized to that of LC3B-I or GAPDH, respectively. Renal tubular cell apoptosis was assessed by TUNEL (e) staining in Con (E1), CIN (E2), CIN+NAC (E3), and CIN+TMP (E4) rats. Semiquantitative analysis of TUNEL staining (f). Figures are representative of 5–8 rats from each group. Data are represented as means ± SD (*n* = 5). ^∗∗^*p* < 0.01 versus Con and ^##^*p* < 0.01 versus CIN.

To verify the induction of renal tubular autophagy in CIN-treated rats, we examined the abundance of the autophagy-associated proteins LC3B and beclin-1 by western blotting. As shown in Figures 3(a) and 3(b), CM treatment significantly increased the conversion of LC3B-I to LC3B-II, resulting in a higher abundance of LC3B-II in CIN-treated rats than in controls. Similar findings were also obtained for the abundance of beclin-1 in CIN-treated rats (Figures 3(a) and 3(d)), while the opposite result was observed for the abundance of p62 (Figures 3(a) and 3(c)).

Meanwhile, renal tubular cells undergoing apoptosis were characterized by condensation of the nuclear chromatin and wrinkling of nuclear membranes (red frame in Figure 2(b)). In addition to TEM, renal tubular apoptosis was further confirmed by a marked increase in the proportion of TUNEL-positive tubular cells in the CIN group (Figures 3(e) and 3(f)). Consistent with our previous findings [14], the glomerulus appeared normal under TEM in CIN-treated kidneys (Figure 2(k)).

Our results indicated that CM might simultaneously induce autophagy and apoptosis in tubular epithelial cells. We next investigated whether CM exposure could also influence the selective degradation of mitochondria through mitophagy using higher magnification TEM, which is regarded as the best approach to provide direct evidence for mitophagy [6]. Concomitantly, as shown in Figure 2(c)–2(g), CM-induced mitophagy (indicated by red frames in the figures) was evident in renal tubular epithelial cells. We found that exposure to CM induced the autophagosomal encapsulation of mitochondria, which confirmed that CM induced mitophagy. Moreover, we observed both the early stage (autophagosome with engulfed mitochondria, Figures 2(c) and 2(d)) and the late stage (single-membrane autolysosomes with residual mitochondria, Figure 2(e)–2(g)) of mitophagy in CIN rats. These results provided strong evidence that CM-induced mitophagy is involved in the pathogenesis of CIN.

Interestingly, treatment with either TMP or NAC markedly reduced the quantity of autophagosomes and the degree of mitophagy (Figures 2(h) and 2(i)), the abundance of LC3B-II and beclin-1 (Figure 3(a)–3(c)), and the frequency of apoptotic cells detected by TUNEL staining (Figures 3 E3, E4 and 3(f)).

### 4.3. TMP Prevented Mitochondrial Fragmentation by Restoring the Alterations in Drp1 and Mfn2 Expression in CIN Rats

**Figure 4:**
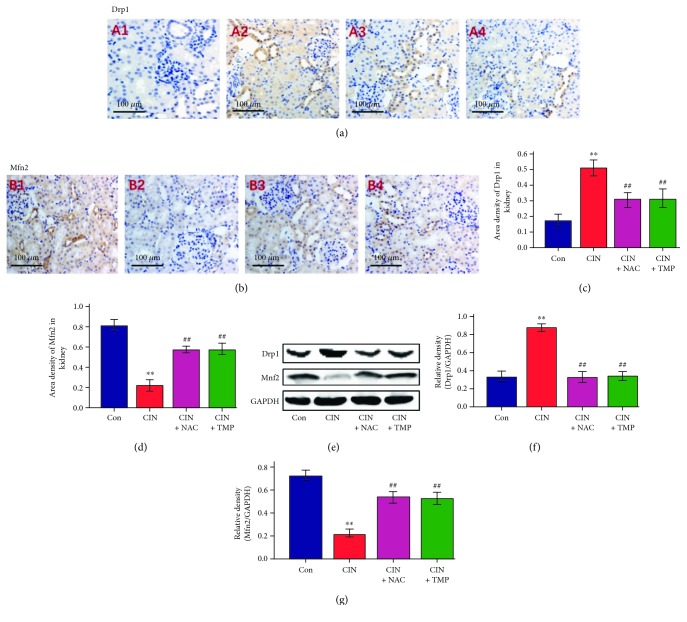
TMP restored mitochondrial dynamic-related protein expression in CIN rats. (a) Drp1 expression was assessed by immunohistochemistry (IHC) in the kidneys of Con (A1), CIN (A2), CIN+NAC (A3), and CIN+TMP (A4) rats (original magnification, ×200). (b) Evaluation of Mfn2 expression in the kidneys of Con (B1), CIN (B2), CIN+NAC (B3), and CIN+TMP (B4) rats by IHC (original magnification, ×200). Semiquantitative analysis of IHC staining for Drp1 (c) and Mfn2 (d). (e) Western blotting analyses of Drp1 and Mfn2 in the kidneys of rats. Relative densitometry analysis of the ratio of Drp1 (f) or Mfn2 (g) to GAPDH. Figures are representative of 5-8 rats from each group. Data are represented as means ± SD (*n* = 5). ^∗∗^*p* < 0.01 versus Con and ^##^*p* < 0.01 versus CIN.

Mitochondria are highly dynamic organelles that undergo frequent fission and fusion. Drp1 and Mfn2 are representative profission and profusion proteins, respectively. The abundance of Drp1 and Mfn2 was evaluated using IHC at 24 h after treatment with CM. As shown in Figures 4(a), A2; 4(b), B2; 4(c); and 4(d), the abundance of Drp1 was remarkably upregulated in the tubules of CIN rats as compared to that of the Con group, while that of Mfn2 was downregulated. Both the TMP and NAC treatments significantly reduced the abundance of Drp1 and restored that of Mfn2 in the kidney as compared with that of the CIN group (Figure 4). Similar changes were demonstrated using western blotting (Figure 4(e)–4(g)). These results suggest that the modulation of MQC might play a role in the renoprotective effect of TMP against CM-induced kidney injury.

### 4.4. TMP Suppressed the Increase in the Abundance of CCL2/CCR2 in Tubular Epithelial Cells of CIN Kidneys

**Figure 5:**
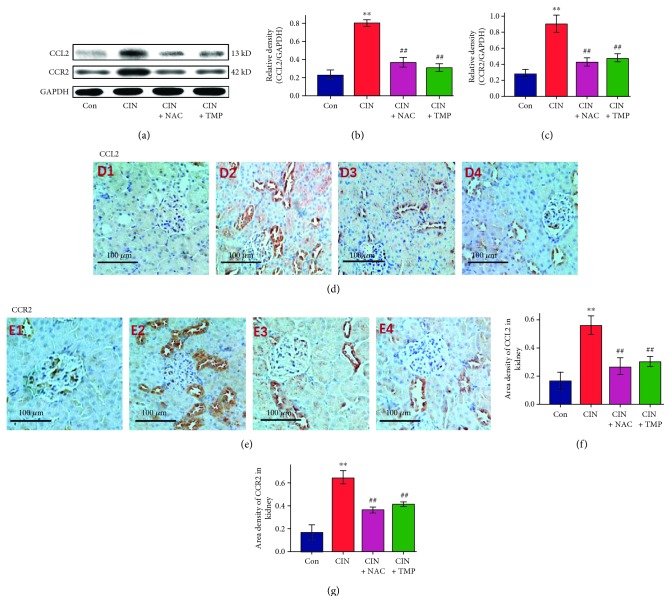
TMP suppressed the increase in the abundance of CCL2 and CCR2 in the tubular epithelial cells of the CIN kidneys. (a) Western blotting analyses of CCL2 and CCR2. Relative densitometry analysis of the ratio of CCL2 (b) or CCR2 (c) to GAPDH. (d) Photomicrographs (original magnification, ×200) illustrate CCL2 staining in the kidney tissues of Con (D1), CIN (D2), CIN+NAC (D3), and CIN+TMP (D4) rats. (e) Photomicrographs (original magnification, ×200) illustrate CCR2 staining in the kidney tissues of Con (E1), CIN (E2), CIN+NAC (E3), and CIN+TMP (E4) rats. Quantitative data for CCL2 (f) or CCR2 (g) staining are graphically presented. Figures are representative of 5–8 rats from each group. Data are represented as means ± SD (*n* = 5). ^∗∗^*p* < 0.01 versus Con and ^##^*p* < 0.01 versus CIN.

We examined the regulation of the CCL2/CCR2 expression by CM in the kidney of CIN rats. As shown in Figure 5(a)–5(c), the abundance of CCL2 and CCR2 was found to be elevated in the kidneys of CIN rats (*p* < 0.01 versus the Con group) by western blotting, while their abundance was suppressed by either TMP or NAC treatment (*p* < 0.01 versus the CIN group).

We next examined the localization of CCL2 and CCR2 in the kidney using immunohistochemistry. As shown in Figure 5, the abundance of both CCL2 (Figure 5(d), D2) and CCR2 (Figure 5(e), E2) was markedly higher in the renal tubules of CIN kidneys than in those of the control kidneys (Figures 5(f) and 5(g), respectively, *p* < 0.01). Furthermore, pretreatment with TMP (Figures 5(d), D4; 5(e), E4; 5(f); and 5(g)) or NAC (Figures 5(d), D3; 5(e), E3; 5(f); and 5(g)) significantly decreased the abundance of CCL2 and CCR2 relative to its abundance in the CIN kidneys (*p* < 0.01).

### 4.5. TMP Suppressed Renal ROS Production and Reduced the Concentrations of IL-6 and TNF-*α* in the Serum and the Kidney

**Figure 6:**
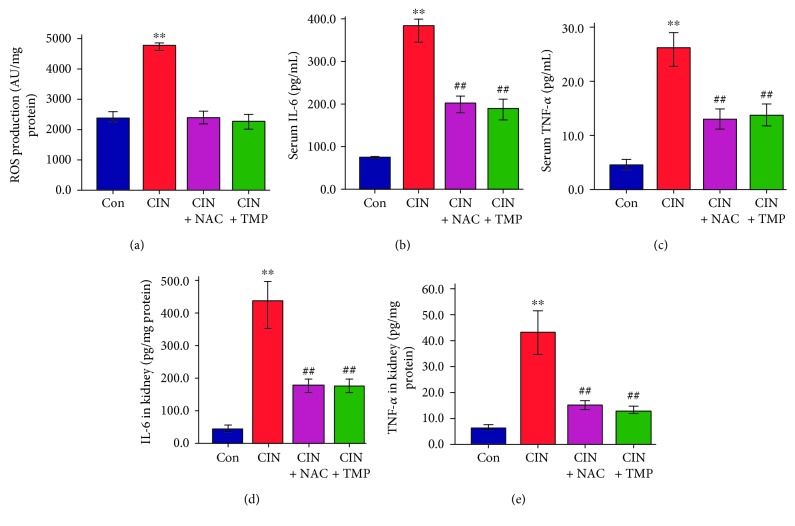
TMP attenuated renal reactive oxygen species production and suppressed contrast medium-induced IL-6 and TNF-*α* upregulation in the serum and kidney. (a) Renal reactive oxygen species production in rats as assessed by the 2′,7′-dichlorofluorescin diacetate method. Concentrations of IL-6 (b) and TNF-*α* (c) in the serum. Abundance of IL-6 (d) and TNF-*α* (e) in the kidney. Data are represented as means ± SD (*n* = 5). ^∗∗^*p* < 0.01 versus Con and ^##^*p* < 0.01 versus CIN.

As shown in Figure 6(a), renal ROS production was increased by 2.1-fold in CIN rats as compared with controls. Conversely, pretreatment with either TMP or NAC notably decreased renal ROS production.

There were significant increases in the concentrations of IL-6 (5.1-fold; *p* < 0.01; Figure 6(b)) and TNF-*α* (5.5-fold; *p* < 0.01; Figure 6(c)) in the serum of CIN rats when compared with those of the controls. Furthermore, enzyme-linked immunosorbent assay analysis showed that CM significantly increased the concentrations of IL-6 and TNF-*α* in the CIN kidneys as compared with those in the control kidneys (*p* < 0.01; Figures 6(d) and 6(e)). Treatment with TMP or NAC significantly decreased the concentrations of IL-6 and TNF-*α* in both the serum and the kidneys of CIN rats (*p* < 0.01; Figure 6).

### 4.6. TMP Prevented Small Intestine Villous Capillary Endothelial Apoptosis and Inflammation after CIN

**Figure 7:**
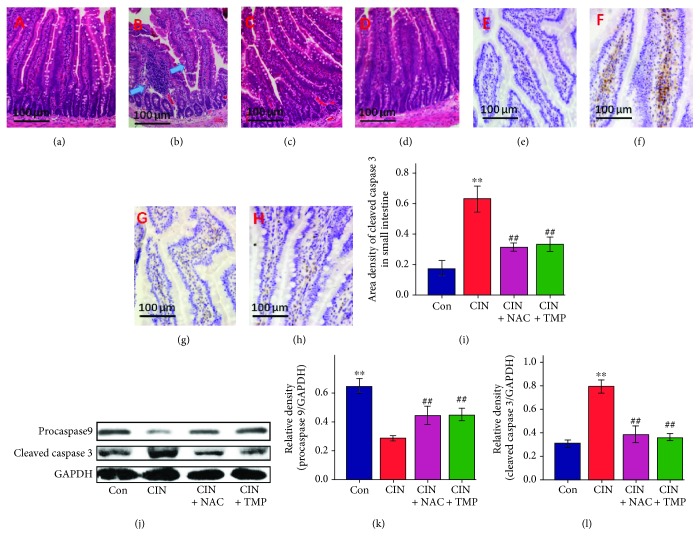
Effects of TMP on the induction of small intestinal villous capillary endothelial apoptosis and inflammation after CIN. Photomicrographs (original magnification, ×200) illustrate hematoxylin and eosin staining of the small intestinal tissues in Con (a), CIN (b), CIN+NAC (c), and CIN+TMP (d) rats (blue arrows indicate the congestion of villous capillaries and the swelling and blunting of villi). Photomicrographs (original magnification, ×200) illustrate cleaved caspase 3 staining in the small intestinal tissues of Con (e), CIN (f), CIN+NAC (g), and CIN+TMP (h) rats. (i) Quantitative data for cleaved caspase 3 staining are graphically presented. (j) Western blotting analyses of procaspase 9 and cleaved caspase 3. Relative densitometry analysis of the ratio of procaspase 9 (k) or cleaved caspase 3 (l) to GAPDH. Figures are representative of 5–8 rats from each group. Data are represented as means ± SD (*n* = 5). ^∗∗^*p* < 0.01 versus Con and ^##^*p* < 0.01 versus CIN.

The underlying mechanism of AKI remains unclear. Recent studies have provided new mechanistic insights and proposed a potential connection between intestinal injury and AKI [24, 35]. Therefore, we next investigated the effects of CM exposure on the small intestine in rats and found obvious signs of intestinal injury, including the congestion of villous capillaries and the swelling and blunting of villi (Figure 7(a)–7(d)). Furthermore, apoptosis of the intestinal villous capillary endothelial cells was demonstrated by an increased abundance of cleaved caspase 3 in CIN rats as detected by IHC and western blotting (Figures 7(f), 7(i), 7(j), and 7(l)). Accordingly, the abundance of procaspase 9 in the intestine was found to be decreased in CIN rats, which further confirmed the occurrence of intestinal cell apoptosis (Figures 7(j) and 7(k)). Interestingly, pretreatment with either TMP or NAC attenuated the intestinal injury as compared with the CIN group (Figure 7).

## 5. Discussion

Despite the introduction of newer and safer CM options, risk stratification for the patient, and additional preventive methods, CIN has become a serious problem in clinical practice and represents the third leading cause of hospital-acquired AKI [1]. At present, no specific drugs or strategies that effectively prevent CIN and could be widely used in clinical practice are available except for extracellular volume expansion [17, 36]. Thus, the development of potential drugs or strategies for CIN prevention and mitigation is badly needed. Our previous studies have shown that TMP has a beneficial effect on CIN by preventing p38 MAPK pathway-mediated renal tubular cell apoptosis under both *in vivo* and *in vitro* conditions [3]. The present data further indicated that the modulation of renal tubular cell autophagy and mitophagy also contributes to the renoprotective effects of TMP against CIN, including the prevention of mitochondrial fragmentation and renal tubular cell apoptosis. Additionally, we further investigated the roles of the proinflammatory CCL2/CCR2 signal and CM-induced small intestinal injury in the pathogenesis of CIN. To our knowledge, this study is the first to demonstrate that renal tubular cell mitophagy, mitochondrial fragmentation, small intestinal injury, and the CCL2/CCR2 signal are critically involved in the pathogenesis of CIN.

TMP showed similar renoprotective effects to 150 mg/kg NAC in this study, including the prevention of histological changes in the kidneys of CIN rats and the induction of the decreases in the levels of urinary AKI biomarkers and Scr, BUN, and CysC. These results are highly consistent with our previous report [3]. As an important mechanism for mediating intracellular homeostasis, autophagy is now regarded as an adaptive response that can be specifically activated by stress or environmental changes [15, 37]. Although it remains controversial whether autophagy has a protective or destructive role in the CIN kidney, work by our laboratory and other investigators has suggested that autophagy contributes to maintaining renal tubule function and protecting against acute tubular injury [14, 37, 38]. The administration of CM resulted in an increase in the quantity of autophagosomes as well as significant elevations in the abundance of LC3B-II and beclin-1 and decreased p62 expression, which further supported that autophagy is involved in the pathological process of CIN and the TMP-dependent renoprotective effect against CIN. The antioxidant and anti-inflammatory functions of TMP as well as its abilities to regulate autophagy and apoptosis were consistent with our previous findings of arsenite-induced nephrotoxicity in HK2 cells [14, 31].

Damaged or dysfunctional mitochondria are regarded as toxic to cells. Therefore, the timely removal of these organelles is critical for the maintenance of cellular viability [6, 39]. Mitophagy, which is a highly selective mechanism for the degradation of mitochondria via autophagy, is critical for maintaining proper cellular functions [39]. There are very limited data on the significance of mitophagy in kidney diseases including CIN [6]. Currently, TEM is regarded as the best method for providing direct evidence of mitophagy. As shown in Figure 2, we demonstrated for the first time that mitophagy was significantly increased in CIN rats, including the early and late stages of mitophagy. These data clearly indicate that mitophagy participates in the pathological mechanism of CIN and might be critical for the maintenance of homeostasis in kidney cells. Notably, the observed increases in mitophagy and mitochondrial fragmentation were accompanied by enhanced renal oxidative stress, aggravated renal histological changes, significantly increased levels of urinary AKI biomarkers and Scr, and higher levels of apoptosis in tubular cells. Thus, these data suggest that oxidative stress and mitochondrial degradation by mitophagy are interrelated in CIN and provide support for the notion that ROS can act as an inducer of autophagy [17]. We thus speculated that mitophagy occurs in tubular cells as a response to the elevation of oxidative stress during the early phase after CM exposure; conversely, when oxidative stress is controlled, mitophagy and autophagy are suppressed. Based on the present data, TMP appeared to suppress renal ROS production and protect tubular cells against CM cytotoxicity via decreased mitophagy, autophagy, and apoptosis. There is increasing evidence to suggest that overproduction of mitochondrial ROS leads to an increase in mitochondrial fragmentation that results in tubular cell damage and apoptosis in diabetic kidney disease (DKD) [12]. Our present data are highly consistent with these findings in DKD. Our results are also consistent with those of other studies that have suggested that the activation of autophagy as a response to mitochondrial pathologies plays essential roles in maintaining renal tubule function and protecting against both acute and chronic kidney diseases [17, 40, 41].

Intracellular MQC includes biogenesis, fusion, fission, and mitophagy. Drp1 plays a key role in mitochondrial fission, whereas Mfn2 is required for mitochondrial fusion (38, 39). As shown in Figure 4, the abundance of Drp1 in CIN rats was significantly higher than that in the control group, while the abundance of Mfn2 was lower. Thus, our data revealed that CM exposure results in enhanced mitochondrial fission and fragmentation as well as mitophagy. Additionally, an abnormal mitochondrial morphology of tubular cells was observed within 24 h after CM exposure, suggesting that CM exposure induces changes in mitochondrial dynamics and intracellular MQC. This finding thus provides new evidence to support the concept that cells can segregate and eliminate dysfunctional mitochondria via intracellular MQC processes. Interestingly, in our study, TMP prevented CM-induced mitochondrial fission and fragmentation. Thus, our present data indicate for the first time that regulating the intracellular MQC of tubular epithelial cells might be a novel mechanism for the renoprotective effects of TMP.

The relationship between inflammation and the pathogenesis of renal damage in CIN has not been fully defined. CCL2, which is a member of the C-C chemokine family, can be produced by many cell types in response to inflammatory stimuli [19, 20]. Previous reports [20] have indicated that renal tubular epithelial cells could act as the initial source of CCL2 in the development of chronic renal damage in a murine renovascular hypertension model. This further suggests a critical role of the inflammatory CCL2 signal in renal disease.

Our previous studies confirmed that the induction of apoptotic cell death in tubular epithelial cells via the p38 MAPK pathway by CM is an important pathogenic mechanism in CIN and that TMP could reverse CM-induced kidney damage by inhibiting the activation of p38 MAPK [3, 5]. Furthermore, p38 MAPK is an important proinflammatory signal regulator, and the p38 MAPK signal cascade is required for the production of various inflammatory cytokines including IFN-*γ*, TNF-*α*, IL-6, and CCL2 [40, 42]. Based on these data, we speculated that the activation of the p38 MAPK pathway after CM exposure could result in an increased production of CCL2 in the kidney. CCL2 activates chemotaxis by binding to specific receptors, and CCR2 is the primary receptor for CCL2 [19]. We thus detected the abundance of CCL2 and CCR2 in the kidneys of the CIN model rats and found that, consistent with our assumption, CM exposure substantially increased their abundance. These findings suggested that the CCL2/CCR2 pathway potentially has a pivotal role in the pathogenesis of CIN.

The abundance of IL-6 and TNF-*α* was substantially higher in the CIN kidneys than in the control kidneys, while pretreatment with either TMP or NAC reversed their upregulation, which further confirmed that inflammation plays a critical role in CIN. Furthermore, TMP and NAC exhibited renoprotective effects against CM-induced AKI by inhibiting the CCL2/CCR2 signal and the subsequent induction of cytokines.

Another novel finding of the current study is that CM exposure led to intestinal injury in addition to CIN and the characteristic lesions included small intestinal villous capillary endothelial apoptosis and inflammation. These findings confirmed again that AKI not only is a disease of the kidney but is also associated with systemic dysfunctions and has numerous complications [22–24]. Interestingly, TMP also showed efficacy in protecting the small intestine against the injuries induced by CM exposure. It is highly probable that its protective effects are closely linked to its antioxidant, anti-inflammatory, and antiapoptotic functions in this model. Our findings are consistent with the preliminary work by Tóth et al. [41], who reported that the intravenous administration of TMP might ameliorate intestinal ischemia-reperfusion injury in rats.

In conclusion, the present study demonstrated novel pathological mechanisms of CIN, including induction of mitophagy, mitochondrial fragmentation, and small intestinal villous capillary endothelial apoptosis. Therefore, targeting damaged mitochondria by activating mitophagy and MQC might be a novel approach for treating CIN. Furthermore, TMP efficiently prevented CM-induced kidney injury *in vivo* by reversing these pathological processes. Mechanistically, TMP efficiently reversed the CM-induced activation of the CCL2/CCR2 pathway, ameliorated renal oxidative stress and aberrant mitochondrial dynamics, and modulated mitophagy in tubular cells. These data provide a novel mechanistic explanation for the renoprotective effects of TMP, and TMP may be a promising therapeutic agent for CIN.

## Data Availability

The data used to support the findings of this study are included within the article.
